# Potential Use of Iron-Limiting Therapy against Cryptococcus neoformans and Effects of Caspofungin on the Host Immune System

**DOI:** 10.1128/msphere.00373-22

**Published:** 2022-08-22

**Authors:** Kazuhiro Itoh, Hiroshi Tsutani, Yasuhiko Mitsuke, Hiromichi Iwasaki

**Affiliations:** a Department of Internal Medicine, National Hospital Organization Awara Hospital, Awara, Japan; b Division of Infection Control and Prevention, University of Fukui Hospital, Fukui, Japan; University of Georgia

**Keywords:** *Cryptococcus neoformans*, iron chelators, spleen tyrosine kinase, caspofungin

## LETTER

We read with interest the article by Moreira-Walsh et al. dealing with the relationship between caspofungin resistance to Cryptococcus neoformans and membrane integrity (1). We would like to present two major points in response to their article regarding the possibility of iron-limiting therapy and compounds that affect membrane permeability that may be used in combination with caspofungin.

Moreira-Walsh et al. concluded that the iron transport impairment caused by the deletion of *CFT1* did not play a major role in the caspofungin resistance of C. neoformans. However, iron limitation is exactly a strategy that is expected to be useful for the treatment of C. neoformans. We hypothesize that the combination of iron chelators and caspofungin may have enhanced the antifungal activity. The novel iron chelator DIBI (denying iron to bacterial infections), in combination with fluconazole, showed a growth inhibition effect against fluconazole-resistant Candida albicans strains (2). The combination of the iron chelator deferoxamine and amphotericin B against C. neoformans showed a fractional inhibitory concentration index of 0.5 and inhibited the growth of C. neoformans (3). In addition, since iron-limiting therapy does not specifically target microorganisms, it does not induce antimicrobial resistance.

There are two problems when compounds that affect cell membrane permeability are used with caspofungin. First, compounds that affect fungal cell membranes may also affect human cell membranes, raising human toxicity issues. This can be expected, since amphotericin B not only targets ergosterol but also weakly binds to cholesterol in animal cells, causing toxicity in the human body (4). This toxicity counteracts caspofungin’s advantage of having low toxicity in humans, as it targets β-d-glucan. The second problem is the bilateral effect of the compounds on the immunoregulation of host immune cells. In our created model of noninfectious inflammation, caspofungin inhibited the activity of splenic tyrosine kinase, which is a key initiator of the immune cascade in fungal infections, and its downstream molecules, thus suppressing the production of inflammatory cytokines and chemokines (5). The inhibition of the splenic tyrosine kinase-mediated pathway is modulatory and is effective for countering the overproduction of inflammatory cytokines, i.e., cytokine storms. On the other hand, the oversuppression of proinflammatory cytokine production may also have a detrimental effect on the prognosis of infectious diseases by oversuppressing host immunity. Host immune suppression is sometimes advantageous to pathogens.

Therefore, it is necessary to verify the effects of the compounds used in combination with caspofungin on host immunity.

We conclude that additional testing with iron chelators is warranted for impairing iron transport as an antifungal treatment strategy. In addition, the concomitant use of compounds that affect cell membrane permeability should be carefully considered, including their toxicity and effects on the host immune system.
